# Thrombocytosis in brachycephalic dogs with brachycephalic obstructive airway syndrome

**DOI:** 10.17221/23/2021-VETMED

**Published:** 2023-02-15

**Authors:** Vladimira Erjavec, Alenka Nemec Svete

**Affiliations:** University of Ljubljana, Veterinary Faculty, Small Animal Clinic, Ljubljana, Slovenia

**Keywords:** brachycephaly, haematology, mean platelet volume, plateletcrit, platelets

## Abstract

Brachycephalic dogs are prone to a conformation-related respiratory disorder known as brachycephalic obstructive airway syndrome (BOAS). Due to its systemic consequences, BOAS should be considered a systemic disease. The aim of the present study was to investigate platelet count (PLT), mean platelet volume (MPV), and plateletcrit (PCT) in patients with various grades of BOAS and non-brachycephalic dogs. The latter served as a control group. We included 106 patients with BOAS and 41 non-brachycephalic dogs. According to the severity of the disease, BOAS patients were classified into grade 1 (17 dogs), grade 2 (42 dogs), and grade 3 (47 dogs). Thrombocytosis was found in 46% (49/106) of all BOAS patients. High platelet mass was found in 62% (66/106) of all BOAS patients. We found significantly (*P* < 0.05) higher PLT, MPV, and PCT in BOAS patients of all grades compared to non-brachycephalic dogs. However, further studies are needed to clarify the role of PLT and platelet indices in BOAS and their relationship with inflammation and hypercoagulability.

Brachycephalic dogs, such as English and French Bulldogs, Pugs, and Boston Terriers, belong to a group of breeds that are prone to a conformation-related respiratory disorder known as brachycephalic obstructive airway syndrome (BOAS) (Packer et al. 2015; Liu et al. 2017). Dogs with BOAS show clinical signs that may include inspiratory dyspnoea, snoring, stertor and stridor, panting, stress, exercise and heat intolerance, cyanosis, and even syncopal episodes, respiratory distress, gastrointestinal problems, and disturbed sleep patterns (Roedler et al. 2013; Dupre and Heidenreich 2016). In addition, BOAS is associated with numerous systemic complications, such as hypertension, hypomagnesaemia, a hypercoagulable state, increased incidence of congenital cardiac abnormalities, acquired myocardial damage, increased concentration of proinflammatory and anti-inflammatory markers, low arterial partial pressure of oxygen and low activity of blood superoxide dismutase (Hendricks et al. 1987; Meola 2013; Rancan et al. 2013; Hoareau and Mellema 2015; Packer et al. 2015; Crane et al. 2017; Erjavec et al. 2021).

Brachycephalic obstructive airway syndrome shares features of obstructive sleep apnoea syndrome (OSAS) (Hendricks et al. 1987; Badran et al. 2014), the most common form of sleep-disordered breathing in humans. Patients with OSAS experience repeated episodes of breathing cessation leading to hypoxia and reoxygenation. These events can lead to oxidative stress, endothelial dysfunction, vascular inflammation, and atherosclerosis, which play an important role in the progression of cardiovascular disease in OSAS patients (Badran et al. 2014; Lavie 2015). Platelets have been proposed as a link between inflammation and the development of cardiovascular complications in OSAS patients (Gabryelska et al. 2018). Platelets play an important role in haemostasis and thrombosis (Ali et al. 2015; Gant et al. 2020). Platelet indicators, routinely evaluated as part of complete blood count analysis, have been suggested as markers of OSAS severity and cardiovascular comorbidity in these patients (Gabryelska et al. 2018). Platelet count (PLT) has been reported in healthy and BOAS-affected brachycephalic dogs (Facin et al. 2020); however, PLT and platelet indices have not been previously reported in brachycephalic dogs with various grades of BOAS. Therefore, our study aimed to evaluate PLT, mean platelet volume (MPV) and plateletcrit (PCT) in canine patients with various grades of BOAS.

## MATERIAL AND METHODS

### Animals

A total of 158 brachycephalic dogs and 84 control dogs were evaluated. Among these, 52 brachycephalic and 43 healthy dogs were excluded from the research for different reasons (concurrent diseases, therapy, BOAS not treated surgically due to owner’s decision, vaccination).

Finally, a total of 106 client-owned dogs diagnosed with BOAS and 41 age-matched, healthy control dogs of non-brachycephalic breeds were included in the study. All BOAS patients were treated surgically for BOAS and control dogs underwent elective surgery.

At the initial presentation, the history of BOAS patients was obtained. All dogs that showed signs of concurrent disease or had received any type of therapy or vaccination within the last month were excluded from the evaluation. A preoperative owner questionnaire [electronic supplementary material (ESM): BOAS questionnaire] was completed for each dog with BOAS to examine a wide range of clinical signs, i.e., respiratory signs, gastrointestinal signs, exercise intolerance, and sleep disorders. The diagnosis of BOAS was based on clinical signs of upper airway obstruction and anatomical anomalies, and disease severity classified as described elsewhere (Dupre and Heidenreich 2016; MacPhail and Fossum 2019). Patients were classified as grade 1, grade 2, and grade 3 based on the decrease in the radius of the airway at the level of the nasopharynx, oropharynx, laryngopharynx, and larynx after soft palate surgery. Grade 1 patients had no or very mild narrowing of the airways. Grade 2 patients had a 50% decrease in airways radius and grade 3 patients had almost complete airway obstruction at the level of the nasopharynx, oropharynx, laryngopharynx, and larynx. The health status of the patients and control dogs was assessed by history, physical examination, and results of routine haematologic (see ESM Table S1, Table S2) and serum routine biochemical analyses (see ESM Table S3).

Formal written informed consent was obtained from the owner. All procedures complied with the relevant Slovenian governmental regulations (Animal Protection Act, Official Gazette of the Republic of Slovenia, 43/2007).

The Ethical Committee on Animal Research of the Veterinary Faculty, University of Ljubljana evaluated and approved the study.

### Blood samples collection and haematological analysis

Venous blood samples were taken from the cephalic vein in all dogs before surgical treatment. Blood samples for complete blood count and white blood cell differential count were collected in tubes containing the anticoagulant EDTA (BD Microtainer; Becton Dickinson, Franklin Lakes, NJ, USA). Haematological analyses were performed within one hour after blood collection using an automated laser-based haematology analyser (ADVIA 120; Siemens, Munich, Germany) equipped with multispecies software.

### Statistical analysis

Data were analysed using commercial software (SPSS v24.0; IBM, Chicago, IL, USA). The Shapiro-Wilk test was performed to test whether the data were normally distributed. Accordingly, parametric (one-way ANOVA with Tukey HSD post-hoc test, independent *t*-test) or non-parametric tests [Kruskal-Wallis test followed by multiple comparisons (*P*-values adjusted for multiple comparisons – Bonferroni correction), Mann-Whitney test] were used to compare parameters between dog groups. The significance level was set at 5%.

## RESULTS AND DISCUSSION

This study is the first to evaluate PLT and platelet indices in brachycephalic dogs with various grades of BOAS. The demographic data of BOAS patients and control dogs are shown in Table 1. We found no significant age differences between BOAS patients and non-brachycephalic dogs; however, the weight of non-brachycephalic dogs was significantly higher than that of BOAS patients. Grade 3 patients were significantly older than grade 1 patients, which may be due to the disease’s progressive nature. Regardless of the grade of BOAS, French Bulldogs were the most common breed (48/106).

**Table 1:** Demographic data of brachycephalic dogs with various grades of brachycephalic obstructive airway syndrome (BOAS) and healthy non-brachycephalic dogs (control)

Demographic characteristics	Control	Grade 1	Grade 2	Grade 3	All patients
Number	41	17	42	47	106
Sex (F/M)	21/20	9/8	16/26	16/31	41/65
Age (months)					
Median	18.0	13.0	31.5	35.0^a^	30.5
IQR	12.5–47.5	9.0–42.0	16.0–60.8	19.0–66.0	16.8–57.0
Weight (kg)					
Median	16.2^b^	9.0	10.0	9.8	9.7
IQR	8.7–27.2	6.8–12.1	8.5–12.6	8.4–11.2	8.4–11.7
Breeds	17 X, 2 LR, 2 AST, 2 COT, 2 SH, 2 ML, 2 BM, 1 MP, 1 GS, 1 WSS, 1 WHT, 1 AT, 1 RWD, 1 RW, 1 DO, 1 STF, 1 DA, 1 CS, 1 HG	10 FB, 4 ST, 1 P, 1 EB, 1 BST	21 FB, 10 BST, 9 P, 1 EB, 1 ST	17 FB, 16 P, 10 BST, 3 EB, 1 ST	48 FB, 26 P, 21 BST, 6 ST, 5 EB

^a^Grade 3 patients significantly (*P* = 0.012) older than grade 1; ^b^Control dogs significantly heavier than grade 1 (*P* = 0.008), grade 3 (*P* = 0.012) and all patients (*P* = 0.000 269)

AST = American Staffordshire Terrier; AT = Airedale Terrier; BM = Bernese Mountain dog; BST = Boston Terrier; COT = Cotton de Tulear; CS = Croatian Sheepdog; DA = Dalmatian; DO = Dobermann; EB = English Bulldog; F = female; FB = French Bulldog; GS = German Spitz; HG = Hungarian Greyhound; IQR = interquartile range (25^th^ to 75^th^ percentile); LR = Labrador Retriever; M = male; ML = Maltese; MP = Miniature Poodle; P = Pugs; RW = Rottweiler; RWD = Romagna Water Dog – Lagotto Romagnolo; SH = Siberian Husky; ST = Shih Tzu; STF = Staffordshire Bull Terrier; WHT = West Highland White Terrier; WSS = White Swiss Shepherd dog; X = mixed breed

In the present study, PLT, MPV, and PCT were significantly higher in BOAS patients than in control dogs (Table 2). When platelet parameters were compared between the three grades of BOAS, PLT (*P* = 0.044) was significantly higher in grade 3 than in grade 1 (Table 2). Contrary to our results, Facin et al. (2020) reported no significant difference in PLT neither between brachycephalic and mesocephalic dogs nor between BOAS-affected brachycephalic dogs and healthy brachycephalic dogs. Thrombocytosis was found in almost half (46%; 49/106) of all BOAS patients. On the other hand, none of the control dogs had PLT above the upper limit of the reference range. Although inflammatory markers were not measured in our study, PLT values that exceeded the upper values of the reference range in our BOAS patients could indicate inflammatory processes (Ali et al. 2015; Woolcock et al. 2017). Very little is known about inflammation in BOAS; however, increased plasma concentrations of pro- and anti-inflammatory cytokines and nitric oxide have been found in dogs with BOAS compared to mesocephalic dogs (Rancan et al. 2013).

**Table 2:** Platelet count and PLT indices of brachycephalic dogs with various grades of the brachycephalic obstructive syndrome (BOAS) and healthy non-brachycephalic dogs (control)

Parameter	Control (*n* = 41)	Grade 1 (*n* = 17)	Grade 2 (*n* = 42)	Grade 3 (*n* = 47)	All patients (*n* = 106)	REF
PLT (10^9^/l)						
Mean ± SD	265 ± 53^a^	341.8 ± 110.2^d^	400.2 ± 82.3	409.6 ± 107.0	395.0 ± 100.4	143–400
No. of dogs	0/41 (0%)	4/17 (25%)	20/42 (48%)	25/47 (53%)	49/106 (46%)	
95% CI	248.4–281.9	285.2–398.5	374.6–425.9	378.2–441.1	375.7–414.4	
MPV (fL)						
Mean ± SD	10.0 ± 1.0^b^	11.4 ± 1.5	11.0 ± 1.6	11.1 ± 1.5	11.1 ± 1.5	7–11
No. of dogs	5/41 (12%)	10/17 (59%)	16/42 (38%)	22/47 (47%)	48/106 (45%)	
95% CI	9.7–10.3	10.6–12.2	10.5–11.4	10.6–11.5	10.8–11.4	
PCT (%)						
Median	0.26^c^	0.35	0.43	0.46	0.43	0.1–0.4
IQR	0.22–0.31	0.31–0.45	0.35–0.50	0.35–0.52	0.34–0.51	
No. of dogs	0/41 (0%)	7/17 (41%)	28/42 (67%)	31/47 (66%)	66/106 (62%)	
95% CI	0.25–0.28	0.32–0.46	0.40–0.47	0.42–0.48	0.41–0.46	

^a^Significantly lower in comparison to grade 1 (*P* = 0.016), grade 2 (*P* < 0.000 1), grade 3 (*P* < 0.000 1) and all patients (*P* < 0.000 1); ^b^Significantly lower in comparison to grade 1 (*P* = 0.004), grade 2 (*P* = 0.010), grade 3 (*P* = 0.002) and all patients (*P* < 0.000 1); ^c^Significantly lower in comparison to grade 1 (*P* = 0.002), grade 2 (*P* < 0.000 1), grade 3 (*P* < 0.000 1) and all patients (*P* < 0.000 1); ^d^Significantly lower in comparison with grade 3 (*P* = 0.044; result of a comparison of three groups of BOAS patients)

95% CI = 95% confidence interval; IQR = interquartile range (25^th^ to 75^th^ percentile); MPV = mean platelet volume; No. of dogs = number of dogs with PLT, MPV or PCT values above the upper value of the reference ranges; PCT = plateletcrit; PLT = platelet count; REF = reference ranges (ADVIA 120; Siemens, Munich, Germany); SD = standard deviation

A number of BOAS patients, which had MPV and PCT above the reference ranges can be seen in Table 2. Mean platelet volume values exceeding the upper value of the reference range could indicate increased PLT activation in BOAS patients, as a consequence of low-grade inflammation and platelet association with a hypercoagulable state (Gant et al. 2020). Hypercoagulable state was not evaluated in our study, but it has been previously reported in severely affected BOAS patients (Crane et al. 2017) and clinically healthy Bulldogs (Hoareau and Mellema 2015).

Mean platelet volume, an indicator of platelet activity (Park et al. 2002), has been shown to increase with OSAS severity and is associated with inflammation and cardiovascular comorbidity in these patients (Gabryelska et al. 2018). It has been reported that the number of nocturnal hypoxaemia periods and the degree of hypoxaemia contribute to platelet activation in OSAS patients (Gabryelska et al. 2018), which may also be the case in our BOAS patients. As reported by Hoareau et al. (2012) BOAS patients also experience hypoxaemia. Plateletcrit indicates the number of circulating platelets in a unit volume of blood, which is analogous to haematocrit for red blood cells. It provides reliable information about the total platelet mass (Woolcock et al. 2017). In our study, 62% (66/106) of all BOAS patients (Table 2) had PCT higher than the upper value of the reference range, indicating increased total platelet mass in BOAS patients.

Our results suggest that platelets may play an important role in BOAS. However, further studies are needed to clarify the role of PLT and platelet indices in BOAS and their relationship with inflammation and hypercoagulability.

## Electronic Supplementary Material

Supplementary Tables
