# Supplementary material for: Thrombocytosis in brachycephalic dogs with brachycephalic obstructive airway syndrome
Source: Vet Med (Praha). 2023 Feb 15;68(2):57–61. doi: 10.17221/23/2021-VETMED (PMC10847823; doi:10.17221/23/2021-VETMED)
Supplement: Supplementary Tables [file VETMED-68-121023-s001.pdf]

https://doi.org/10.17221/23/2021-VETMED

Thrombocytosis in brachycephalic dogs with brachycephalic obstructive airway syndrome

*Vladimira Erjavec, Alenka Nemec Svete**

University of Ljubljana, Veterinary Faculty, Small Animal Clinic, Ljubljana, Slovenia

**Corresponding author: alenka.nemecsvete@vf.uni-lj.si*

The authors are fully responsible for both the content and the formal aspects of the electronic supplementary material. No editorial adjustments were made.

Electronic Supplementary Material (ESM)

Table S1. Haematological parameters of brachycephalic dogs with various grades of brachycephalic obstructive airway syndrome (BOAS) and healthy non-brachycephalic dogs (control)

Table S2. White blood cell count and white blood cell differential count of brachycephalic dogs with various grades of brachycephalic obstructive airway syndrome (BOAS) and healthy non-brachycephalic dogs (control)

Table S3. Biochemical parameters of brachycephalic dogs with various grades of brachycephalic obstructive airway syndrome (BOAS) and healthy non-brachycephalic dogs (control)

https://doi.org/10.17221/23/2021-VETMED

Table S1. Haematological parameters of brachycephalic dogs with various grades of brachycephalic obstructive airway syndrome (BOAS) and healthy non-brachycephalic dogs (control)

Parameter	Control (n = 41)	Grade 1 (n = 17)	Grade 2 (n = 42)	Grade 3 (n = 47)	All patients (n = 106)	REF
RBC (10^{12}/l)						
Mean ± SD	7.1 ± 0.7	7.2 ± 0.9	7.1 ± 0.8	6.9 ± 0.8	7.1 ± 1.0	5.7–8.8
HGB (g/l)						
Mean ± SD	168.4 ± 16.2	170.4 ± 19.8	172.7 ± 24.8	166.4 ± 22.4	169.6 ± 23.0	129–184
HCT (l/l)						
Mean ± SD	0.494 ± 0.050	0.496 ± 0.061	0.504 ± 0.072	0.493 ± 0.069	0.498 ± 0.069	0.37–0.57
MCV (fL)						
Mean ± SD	69.6 ± 3.4	69.0 ± 2.4	70.6 ± 3.1	70.6 ± 2.7	70.3 ± 2.9	58.8–71.2
MCH (pg)						
Mean ± SD	23.6 ±1.2	23.7 ± 0.9	24.2 ± 0.8	24.2 ± 0.7	24.1 ± 0.8	20.5–24.2
MCHC (g/l)						
Mean ± SD	341.0 ± 8.8	343.0 ± 6.2	342.8 ± 8.4	342.5 ± 7.5	342.7 ± 7.6	320–360

HCT = haematocrit; HGB = haemoglobin concentration; MCH = mean corpuscular haemoglobin; MCHC = mean corpuscular haemoglobin concentration; MCV = mean corpuscular volume; RBC = red blood cell count; REF = reference ranges (ADVIA 120; Siemens, Munich, Germany); SD = standard deviation

https://doi.org/10.17221/23/2021-VETMED

Table S2. White blood cell count and white blood cell differential count of brachycephalic dogs with various grades of brachycephalic obstructive airway syndrome (BOAS) and healthy non-brachycephalic dogs (control)

Parameter	Control (n = 37)	Grade 1 (n = 17)	Grade 2 (n = 42)	Grade 3 (n = 47)	All patients (n = 106)	REF
WBC (10^9/l)						
Median	9.6	9.2	10.1	10.2	10.1	5.2–13.9
IQR	8.7–11.3	8.1–11.5	8.5–12.8	8.1–13.3	8.2–12.8	
NEUT (10^9/l)						
Median	5.26	6.21	6.62	6.12	6.32	3.9–8.0
IQR	4.30–7.16	4.71–6.95	5.22–8.04	4.79–7.84	4.99–7.80	
LYMPH (10^9/l)						
Median	2.85	2.37	2.48	2.44	2.45	1.3–4.1
IQR	2.36–3.39	1.91–2.93	1.95–3.32	1.81–2.98	1.95–3.02	
MONO (10^9/l)						
Median	0.380	0.500	0.540	0.530	0.530	0.2–1.1
IQR	0.305–0.470	0.360–0.650	0.413–0.778	0.400–0.690	0.388–0.695	
EOS (10^9/l)						
Median	0.480	0.260	0.255	0.280	0.280	0.0–0.6
IQR	0.300–0.750	0.125–0.565	0.178–0.445	0.150–0.440	0.170–0.443	
BASO (10^9/l)						
Median	0.020	0.020	0.020	0.020	0.020	0.0–0.1
IQR	0.020–0.030	0.010–0.040	0.018–0.033	0.020–0.030	0.020–0.030	
LUC (10^9/l)						
Median	0.020	0.020	0.025	0.030	0.020	0.0–0.3
IQR	0.015–0.030	0.015–0.030	0.010–0.050	0.010–0.060	0.010–0.040	

BASO = basophil count; EOS = eosinophil count; LUC = large unstained cell count; LYMPH = lymphocyte count; MONO = monocyte count; NEUT = neutrophil count; REF = reference ranges (ADVIA 120; Siemens, Munich, Germany); SD = standard deviation; WBC = white blood cell count

https://doi.org/10.17221/23/2021-VETMED

Table S3. Biochemical parameters of brachycephalic dogs with various grades of brachycephalic obstructive airway syndrome (BOAS) and healthy non-brachycephalic dogs (control)

Number	Control (n = 14)	Grade 1 (n = 17)	Grade 2 (n =42)	Grade 3 (n = 47)	All patients (n = 106)	REF
Glucose (mmol/l)						
Median	5.60	6.00	5.85	5.90	5.90	3.61–6.55
IQR	5.38–6.23	5.60–6.20	5.40–6.53	5.40–6.50	5.40–6.42	
Urea (mmol/l)						
Median	7.46	5.69	6.00	6.20	6.07	2.50–9.60
IQR	5.12–8.59	4.80–6.89	5.07–6.92	4.86–7.34	4.95–7.21	
Creatinine (μmol/l)						
Median	90.2	83.1	84.9	84.9	86.6	44.2–132.6
IQR	84.5–135.4	70.2–116.9	71.7–105.9	70.2–102	71.1–107.3	
ALP (μkat/l)						
Median	0.420	0.808	0.573	0.560	0.607	0.333–2.550
IQR	0.275–0.627	0.537–1.537	0.385–0.808	0.383–0.807	0.385–0.890	
ALT (μkat/l)						
Median	0.620	0.762	0.790	0.807	0.795	0.350–2.467
IQR	0.377–0.807	0.610–1.087	0.565–1.103	0.593–0.958	0.593–1.085	

ALP = alkaline phosphatase; ALT = alanine aminotransferase; IQR = interquartile range (25th to 75th percentile); REF = own reference ranges
